# Neurophysiological Features of Tremor during Walking in Parkinson's Disease

**DOI:** 10.1002/mdc3.14293

**Published:** 2024-12-03

**Authors:** Matteo Costanzo, Francesco Marchet, Giorgio Leodori, Carolina Cutrona, Maria Ilenia De Bartolo, Giorgio Vivacqua, Antonella Conte, Giovanni Fabbrini, Alfredo Berardelli, Daniele Belvisi

**Affiliations:** ^1^ Department of Human Neuroscience Sapienza University of Rome Rome Italy; ^2^ Department of Neuroscience Istituto Superiore di Sanità Rome Italy; ^3^ Department of Neurology and Clinical Neurophysiology, IRCCS Neuromed Pozzilli Italy; ^4^ Department of Experimental Morphology and Microscopy‐Integrated Research Center (PRAAB) Campus Biomedico University of Rome Rome Italy

**Keywords:** Tremor during walking, Parkinson's disease, Tremor, Neurophysiology, Gait

## Abstract

**Background:**

In Parkinson's Disease (PD), upper limb tremor during walking (TW) is observed and clinical observations suggest it may represent a variant of rest tremor. However, its neurophysiological characteristics remain unexplored.

**Objectives:**

This study compared the neurophysiological features of TW with other PD tremors and tested whether TW arises from reduced ipsilateral arm swing.

**Methods:**

Inertial measurement units were used to measure frequency and amplitude of tremors and arm swing during walking in 25 PD patients.

**Results:**

TW shared a similar frequency with rest and re‐emergent tremor (RET) but showed significantly greater amplitude. A positive correlation was observed between the amplitude and frequency of TW with those of rest and RET on the same side. TW distribution was unrelated to reduced arm swing during walking, suggesting TW is not due to decreased ipsilateral arm movement.

**Conclusions:**

These findings suggest that walking may act as a provocation maneuver, triggering rest tremor.

Tremor in Parkinson's disease (PD) manifests in various clinical forms, including rest, re‐emergent (RET), postural and kinetic tremor.^1^, ^2^, ^3^, ^4^ Upper limb tremor also appears during walking^5^ and a recent clinical study on 51 PD patients demonstrated that tremor during walking (TW) was similar to rest tremor in body distribution, severity, and treatment response.^5^ The observation that TW occurs more frequently on the more affected side of patients suggests that arm swing reduction during walking may induce a condition of stability, promoting TW emergence.^5^ Only one study employed accelerometric recordings to assess amplitude and frequency of TW in PD, and the authors found higher tremor amplitude and frequency during walking compared to rest.^6^ However, measurements were restricted to the most affected side for 5 seconds, without arm swing quantification, limiting information on TW pathophysiology.

The aim of the study was to characterize the neurophysiological features by quantitatively assessing amplitude and frequency of TW on both sides, comparing TW with other PD tremors. By using an inertial measurement unit (IMU), we measured frequency and amplitude of tremor at rest, while maintaining an outstretched arm position and during walking in 25 PD patients. Moreover, we quantified the amplitude of arm swing during walking to investigate whether TW emergence is linked to reduced ipsilateral arm pendular movement.

## Methods

Twenty‐five idiopathic PD patients (9F, 16 M) were enrolled from the Movement Disorders outpatient clinic of Sapienza University of Rome and IRCCS Neuromed. The 30 minutes experimental session was conducted after 24‐hours withdrawal from dopaminergic treatment, with patients evaluated OFF‐medication. Demographic data were collected. Disease stage and motor symptoms severity were assessed using Hoehn and Yahr scale and MDS‐sponsored revision of the Unified Parkinson's Disease Rating Scale part III, respectively.^7^, ^8^ Neurophysiological tremor and pendular movements assessment were conducted using a wearable wireless gyroscope (BWT901CL IMU; WitMotion Shenzhen Co., Ltd, China) (Fig. S1). Tremor was evaluated at rest, with outstretched arms and during walking. Tremor analysis employed three‐dimensional gyroscope data, processed via Fast Fourier Transform (FFT) to compute power spectral density, determining tremor frequency and amplitude across axes^9^, ^10^, ^11^, ^12^ (Fig. S2). The mean peak frequency and mean amplitude of tremors were averaged from both hands for each patient.

Detailed description of methodological procedures and experimental paradigm are reported in Supplementary materials.

### Statistical Analysis

We used Prism Graph‐Pad version 10.1.1 (Boston, MA, USA). Continuous variables are reported as means ± standard deviations. The Shapiro–Wilk test assessed distribution of data. Parametric or non‐parametric tests were used to evaluate differences in terms of frequency and amplitude between tremors. Spearman rank correlation was used to analyze correlations between tremor frequencies and amplitudes (within and between sides) as well as between the mean amplitudes or mean peak frequencies of the tremors and arm swing amplitude. *P* values < 0.05 were considered significant, with Tukey correction applied for multiple comparisons.

## Results

Demographic, clinical characteristics and body distribution of tremors are described in detail in Supplementary materials.

### Tremor Frequency Analysis

**Figure 1:**
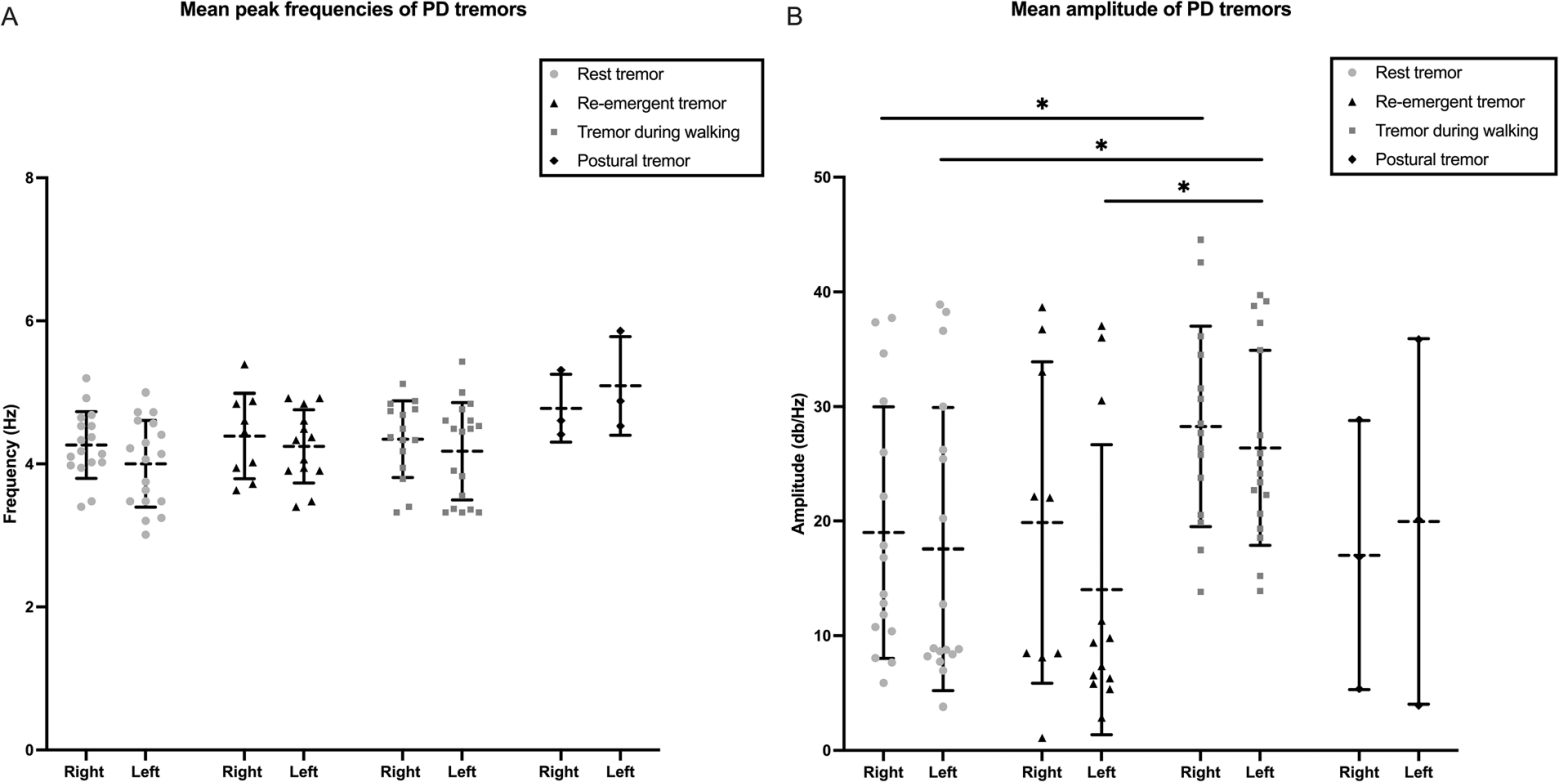
Amplitude and frequency of different types of tremors. (A) Comparison of frequency, expressed in Hz, across different types of tremors. (B) Comparison of amplitude, expressed in dB/Hz, across different types of tremors. Dotted lines represent the mean values, while error bars indicate standard deviation. The asterisk (*) indicates statistical significance.

The overall mean peak frequencies of different tremors are reported in Table 1. A two‐way repeated measure ANOVA, showed a significant effect of TREMOR TYPE (F (3,44) = 5.651, *P* = 0.0023), but no significant effect for SIDE (F (1,44) = 0.2840, *P* = 0.5968) or their interaction (F (3,44) = 1.422, *P* = 0.2490). The post‐hoc analysis did not reveal any significant differences (Fig. 1A). Spearman's correlation coefficient showed a positive correlation between mean peak frequencies of rest tremor and those of TW (r = 0.512; *P* = 0.04) and RET (r = 0.819; *P* = 0.007), all on the right side. On the left side, a positive correlation was found between the mean peak frequencies of rest tremor and those of TW (r = 0.860; *P* = 0.000005) and RET (r = 0.811; *P* = 0.000766). The mean peak frequencies of TW were positively correlated with those of RET (r = 0.797; *P* = 0.001).

**TABLE 1:** Neurophysiological characteristics of different types of tremors in PD patients

Tremor type	Mean peak frequency (Hz)	Mean amplitude (db/Hz)	Right side		Left side	
			Mean peak frequency (Hz)	Mean amplitude (db/Hz)	Mean peak frequency (Hz)	Mean amplitude (db/Hz)
Rest tremor	4.1 ± 0.6	18.3 ± 11.6	4.3 ± 0.5	18.3 ± 11	4 ± 0.6	18.2 ± 12.5
Re‐emergent tremor	4.3 ± 0.6	16.6 ± 13.2	4.4 ± 0.6	19.8 ± 14	4.2 ± 0.5	14 ± 12.7
Tremor during walking	4.3 ± 0.6	27.3 ± 8.5	4.4 ± 0.5	28.3 ± 8.8	4.2 ± 0.7	26.4 ± 8.5
Postural tremor	4.9 ± 0.6	18.5 ± 16.3	4.8 ± 0.5	17.1 ± 16.6	5.1 ± 0.7	19.9 ± 22.6

### Amplitude of Different Types of Tremors

The global mean amplitudes of different tremors are reported in Table 1. A two‐way repeated measure ANOVA showed a significant effect of TREMOR TYPE (F (3,50) = 21.61, *P* < 0.0001), but no significant effect for SIDE (F (1,32) = 0.003543, *P* = 0.9529) or their interaction (F (3,50) = 0.8444, *P* = 0.4761). Post‐hoc analysis showed significant amplitude differences between rest and TW on both sides, with rest tremor showing lower amplitudes on the right side (*P* = 0.0008) and on the left side (*P* = 0.0065). RET amplitude was lower than TW on the left side (*P* = 0.0014) (Fig. 1B). Spearman's correlation coefficient showed a positive correlation between mean amplitude of rest tremor and that of TW (r = 0.725; *P* = 0.002212) and RET (r = 0.963; *P* = 0.000032), as well as between mean amplitude of TW and that of the RET (r = 0.733; *P* = 0.02) all on the right side. On the left side, a positive correlation was found between the mean amplitude of resting tremor and those of TW (r = 0.739; *P* = 0.001) and RET (r = 0.921; *P* = 0.00002). The mean amplitude of TW was positively correlated with that of RET (r = 0.723; *P* = 0.008).

### Relationship between TW and Arm Swing

In cases of unilateral TW, tremor occurred on the side with the lesser pendular movement in 7 patients, and on the opposite side in 10 patients (Table S1). We also observed no correlation between the neurophysiological features of TW and the amplitude or frequency of arm swing (Fig. S3).

## Discussion

The novelty of our study lies in the neurophysiological characterization of TW in PD patients. We observed that TW shares similar frequency with rest and RET, suggesting a common pathophysiological mechanism. Our findings align well with our previous clinical study, hypothesizing that TW might be a clinical variant of rest tremor, based on similar body distribution and treatment response.^5^ Our results contrast with a previous study reporting higher TW frequency than rest tremor in PD patients.^6^ However, in that paper^6^ tremor recording, limited to 5 seconds, might have been likely insufficient to accurately characterize tremor frequency. In the patients here studied we found a significant higher TW amplitude compared to rest and re‐emergent tremors, corroborating previous evidence.^6^ In the context of parkinsonian tremor, the frequency of rest tremor is quite stable over time and primarily dependent on synchronized activity of central oscillators within the basal ganglia thalamic circuits,^13^, ^14^ while the amplitude is more variable and is believed to be modulated by cortico‐cerebello‐thalamic circuits, acting as a “dimmer”.^14^ Amplitude is also modulated by stress and cognitive load.^15^

The relationship between movement and tremor represents an intriguing aspect of PD. Voluntary movement triggers action tremor and suppresses resting and re‐emergent tremor.^3^, ^16^ Walking is an activity involving volitional phases, which includes initiating movements, and automatic phases, including the rhythmic arm swinging and lower limbs walking cycle.^17^, ^18^ Neuronal networks involved in motor planning, including several regions such as parietal, cerebellar, premotor, and supplementary motor areas are activated during gait.^19^ Walking could activate circuits in common with those involved in tremor.^20^ It may modulate cerebello‐thalamic‐cortical circuits, though further studies are needed to confirm this possibility. Alternatively, the arm's mechanical, inertial and elastic characteristics, being free to move and governed only by gravity might also contribute to the increased TW amplitude.

We observed a significant correlation between the amplitudes and frequencies of TW, rest and RET tremor on the same side of the body, but not across different sides of the body. This aligns with the hypothesis of multiple oscillators generating tremor in different extremities of PD patients, as shown by previous studies using cross‐spectral analysis of tremor activity in affected muscles.^21^ Our observation could, on one hand, corroborate this hypothesis and, on the other hand, provide proof that the same central oscillator is involved in the genesis of resting, RET, and TW on the same side of the body.

We failed to confirm our hypothesis that TW is due to reduced pendular arm movement. Two hypotheses, not mutually exclusive, can be considered. Firstly, the reduction in arm swinging during walking in PD patients might not represent a condition of motor stability, where tremulous muscles are relaxed.^3^, ^22^ However, from a biomechanical perspective, several studies have suggested that impaired arm swing may stem from axial rigidity or the inappropriate timing in the activation of shoulder flexors or extensors,^23^, ^24^, ^25^ thus involving more proximal muscles rather than the hands, where TW manifests. Alternatively, the neurophysiological circuits underlying TW and arm swing could differ. Although parkinsonian tremors are typically believed to arise from oscillatory activity within dopamine‐depleted subthalamic nucleus (STN)/internal globus pallidus (GPi) that travel across a cerebello‐talamo‐cortical pathway,^14^, ^26^ clinical and neurophysiological studies seem to suggest that the STN and related dopaminergic systems have a relatively weaker influence on the executive structures involved in the arm swing.^27^, ^28^

We took several precautions to avoid confounding factors. PD patients were diagnosed based on current guidelines^29^ and evaluated in “off” medication state to eliminate treatment effects. We used IMUs with a 200 Hz sampling rate, suitable for the typical PD tremor frequency of 4–12 Hz, and limited recording times to 60 seconds to minimize fatigue, ensuring tremor assessment accuracy.^30^ This duration is considered adequate to capture tremor parameters.^12^, ^30^ IMU data were processed according to established guidelines.^12^

We recognize several limitations. The absence of simultaneous evaluation of surface electromyographic activity is one, even if for describing the main movement characteristics of tremor, the use of accelerometers or gyroscopes is sufficient.^12^ Moreover, although we observed a trend of higher frequency of postural tremor, the small number of PD patients with postural tremor in our cohort may have affected our ability to detect statistically significant differences between groups.

In conclusion, we described the neurophysiological features of TW in PD patients. Our findings suggest that walking may act as a provocation maneuver, revealing rest tremor in the upper limbs. The observation that the amplitude of TW is larger compared to other types of parkinsonian tremors may be significant from a clinical point of view. Specifically, asking a patient to walk can unveil the presence of a rest tremor not otherwise observable. Future studies could provide a more detailed understanding of the processes involved in this tremor form.

## Disclosures

**Ethical Compliance Statement:** The study was conducted according to the guidelines of the Declaration of Helsinki and approved by the local institutional review board (Sapienza University of Rome Ethics Committee, No. 5830). All patients were informed of the study purpose and written informed consent was obtained from all participants. We confirm that we have read the Journal's position on issues involved in ethical publication and affirm that this work is consistent with those guidelines.

**Funding Sources and Conflict of Interest:** The authors report no potential conflict of interest related to the research in this article. This research did not receive any specific grant from funding agencies in the public, commercial or not‐for‐profit sectors.

**Financial Disclosures for the Previous 12 Months:** The authors declare that there are no disclosures to report.

## Authors Roles

(1) Research project: A. Conception, B. Organization, C. Execution; (2) Statistical Analysis: A. Design, B. Execution, C. Review and Critique; (3) Manuscript Preparation: A. Writing of the first draft, B. Review and Critique.

M.C.: 1A, 1B, 1C, 2A, 2B, 2C, 3A

F.M.: 1A, 1B, 1C, 2B, 3A

G.L.: 1A; 1B, 1C, 2A, 2B

C.C.: 1A, 1B, 1C, 2A

M.I.B.: 1A, 1C, 2A

G.V.: 1C, 2A, 3B

A.C.: 1A, 1B, 2A, 2C, 3B

G.F.: 1A, 1B, 2A, 2C, 3B

A.B.: 1A, 1B, 2A, 2C, 3B

D.B.: 1A, 1B, 2A, 2B, 2C, 3A, 3B

## Supporting information


**Figure S1.** Experimental paradigm. Panel A shows the Inertial Mesurement Unit used in the study (BWT901CL Inertial Measurement Unit by WitMotion Shenzhen Co., Ltd, China). Panel B and C show the anatomical localization of the accelerometer axes for the assessment of tremor on the dorsal side of the hand (B) and on the mid‐deltoid (C). The lower part of the figure illustrates the experimental paradigm, with the conditions used to evaluate rest tremor (D), re‐emergent and postural tremor (E) and tremor during walking (F).

**Figure S2.** Tremor analysis: raw data and Fast Fourier Transform. Representative data displaying raw measurements (A) and Fast Fourier Transform analysis (B) for a single subject, illustrating conditions of rest tremor, re‐emergent tremor, and tremor during walking.

**Figure S3.** Correlation matrix. The coefficients of correlations between tremor and arm swing parameters (frequency and amplitude) are reported. *Abbreviations*: RT, resting tremor; PT, postural tremor; RET, re‐emergent tremor; TW, tremor during walking; freq, frequency; amp, amplitude; AS, arm swing.

**Table S1.** Neurophysiological characteristics of arm swing movements.

**Data S1.** Additional details on methods, materials, and results are provided.

## Data Availability

Data are available upon reasonable request to the corresponding author.

## References

[1] Belvisi D , Conte A , Bologna M , et al. Re‐emergent tremor in Parkinson's disease. Parkinsonism Relat Disord 2017;36:41–46. 28007517 10.1016/j.parkreldis.2016.12.012

[2] Bhatia KP , Bain P , Bajaj N , et al. Consensus statement on the classification of tremors. From the task force on tremor of the International Parkinson and Movement Disorder Society. Mov Disord 2018;33(1):75–87. 29193359 10.1002/mds.27121 PMC6530552

[3] Dirkx MF , Zach H , Bloem BR , Hallett M , Helmich RC . The nature of postural tremor in Parkinson disease. Neurology 2018;90(13):e1095–e1103. 29476038 10.1212/WNL.0000000000005215 PMC5880634

[4] Belvisi D , Conte A , Cutrona C , Costanzo M , Ferrazzano G , Fabbrini G , Berardelli A . Re‐emergent tremor in Parkinson's disease: the effect of dopaminergic treatment. Eur J Neurol 2018;25(6):799–804. 29512863 10.1111/ene.13619

[5] Costanzo M , Cutrona C , Leodori G , et al. Distal upper limb tremor during walking in Parkinson's disease. Mov Disord Clin Pract 2023;10(8):1198–1202. 37635779 10.1002/mdc3.13814 PMC10450241

[6] Uchida K , Hirayama M , Yamashita F , Hori N , Nakamura T , Sobue G . Tremor is attenuated during walking in essential tremor with resting tremor but not parkinsonian tremor. J Clin Neurosci 2011;18(9):1224–1228. 21745741 10.1016/j.jocn.2010.12.053

[7] Hoehn MM , Yahr MD . Parkinsonism: onset, progression and mortality. Neurology 1967;17(5):427–442. 6067254 10.1212/wnl.17.5.427

[8] Antonini A , Abbruzzese G , Ferini‐Strambi L , et al. Validation of the Italian version of the Movement Disorder Society—unified Parkinson's disease rating scale. Neurol Sci 2013;34(5):683–687. 22678179 10.1007/s10072-012-1112-z

[9] Bartolić A , Šantić M , Ribarič S . Automated tremor amplitude and frequency determination from power spectra. Comput Methods Programs Biomed 2009;94(1):77–87. 19081159 10.1016/j.cmpb.2008.10.007

[10] Martinez Manzanera O , Elting JW , Van Der Hoeven JH , Maurits NM . Tremor detection using parametric and non‐parametric spectral estimation methods: a comparison with clinical assessment. PLoS One 2016;11(6):e0156822. 27258018 10.1371/journal.pone.0156822 PMC4892538

[11] Timmer J , Lauk M , Deuschl G . Quantitative analysis of tremor time series. Electroencephalogr Clin Neurophysiol 1996;101(5):461–468. 8913201

[12] Vial F , Kassavetis P , Merchant S , Haubenberger D , Hallett M . How to do an electrophysiological study of tremor. Clin Neurophysiol Pract 2019;4:134–142. 31886436 10.1016/j.cnp.2019.06.002 PMC6923291

[13] Helmich RC , Hallett M , Deuschl G , Toni I , Bloem BR . Cerebral causes and consequences of parkinsonian resting tremor: a tale of two circuits? Brain 2012;135(11):3206–3226. 22382359 10.1093/brain/aws023 PMC3501966

[14] Helmich RC , Toni I , Deuschl G , Bloem BR . The pathophysiology of essential tremor and Parkinson's tremor. Curr Neurol Neurosci Rep 2013;13(9):378. 23893097 10.1007/s11910-013-0378-8

[15] Dirkx MF , Zach H , Van Nuland AJ , Bloem BR , Toni I , Helmich RC . Cognitive load amplifies Parkinson's tremor through excitatory network influences onto the thalamus. Brain 2020;143(5):1498–1511. 32355951 10.1093/brain/awaa083

[16] Leodori G , De Bartolo MI , Fabbrini A , Costanzo M , Mancuso M , Belvisi D , et al. The role of the motor cortex in tremor suppression in Parkinson's disease. J Parkinson's Dis 2022;12(6):1957–1963. 35811537 10.3233/JPD-223316

[17] Jahn K , Deutschländer A , Stephan T , et al. Supraspinal locomotor control in quadrupeds and humans. Prog Brain Res 2008;171:353–362. 18718326 10.1016/S0079-6123(08)00652-3

[18] Takakusaki K . Neurophysiology of gait: from the spinal cord to the frontal lobe. Mov Disord 2013;28(11):1483–1491. 24132836 10.1002/mds.25669

[19] Takakusaki K . Functional neuroanatomy for posture and gait control. J Mov Disord 2017;10(1):1–17. 28122432 10.14802/jmd.16062 PMC5288669

[20] Sciacca G , Giliberto C , Luca A , Nicoletti A , Zappia M . Seated man walking: a provocation maneuver for parkinsonian tremor. Mov Disord Clin Pract 2016;3(2):212–213. 30713918 10.1002/mdc3.12260 PMC6353439

[21] Raethjen J , Lindemann M , Schmaljohann H , Wenzelburger R , Pfister G , Deuschl G . Multiple oscillators are causing parkinsonian and essential tremor. Mov Disord 2000;15(1):84–94. 10634246 10.1002/1531-8257(200001)15:1<84::aid-mds1014>3.0.co;2-k

[22] Swinnen BEKS , De Bie RMA , Hallett M , Helmich RC , Buijink AWG . Reconstructing Re‐emergent tremor. Mov Disord Clin Pract 2023;10(9):1293–1296. 37772284 10.1002/mdc3.13806 PMC10525057

[23] Buchthal F , Fernandez‐Ballesteros ML . Electromyographic study of the muscles of the upper arm and shoulder during walking in patients with parkinson's disease. Brain 1965;88(5):875–896. 5864465 10.1093/brain/88.5.875

[24] Franzén E , Paquette C , Gurfinkel VS , Cordo PJ , Nutt JG , Horak FB . Reduced performance in balance, walking and turning tasks is associated with increased neck tone in Parkinson's disease. Exp Neurol 2009;219(2):430–438. 19573528 10.1016/j.expneurol.2009.06.013 PMC2775914

[25] Wright WG , Gurfinkel VS , Nutt J , Horak FB , Cordo PJ . Axial hypertonicity in Parkinson's disease: direct measurements of trunk and hip torque. Exp Neurol 2007;208(1):38–46. 17692315 10.1016/j.expneurol.2007.07.002 PMC2144734

[26] Wilken M , Andres DS , Bianchi G , Hallett M , Merello M . Persistence of basal ganglia oscillatory activity during tremor attenuation by movement in Parkinson's disease patients. Mov Disord 2024;39(5):768–777. 10.1002/mds.29679 38415321

[27] Warmerdam E , Romijnders R , Hansen C , et al. Arm swing responsiveness to dopaminergic medication in Parkinson's disease depends on task complexity. npj Parkinson's Dis 2021;7(1):89. 34611152 10.1038/s41531-021-00235-1 PMC8492858

[28] Crenna P , Carpinella I , Lopiano L , et al. Influence of basal ganglia on upper limb locomotor synergies. Evidence from deep brain stimulation and L‐DOPA treatment in Parkinson's disease. Brain 2008;131(12):3410–3420. 18952669 10.1093/brain/awn272

[29] Postuma RB , Berg D , Stern M , et al. MDS clinical diagnostic criteria for Parkinson's disease: MDS‐PD Clinical Diagnostic Criteria. Mov Disord 2015;30(12):1591–1601. 26474316 10.1002/mds.26424

[30] Elble RJ , McNames J . Using portable transducers to measure tremor severity. Tremor Other Hyperkinetic Mov 2016;6:375. 10.7916/D8DR2VCC PMC4872171 27257514

