# Supplementary figures and images for: Neurophysiological Features of Tremor during Walking in Parkinson's Disease
Source: Mov Disord Clin Pract. 2024 Dec 3;12(2):226–30. doi: 10.1002/mdc3.14293 (PMC11802656; doi:10.1002/mdc3.14293)

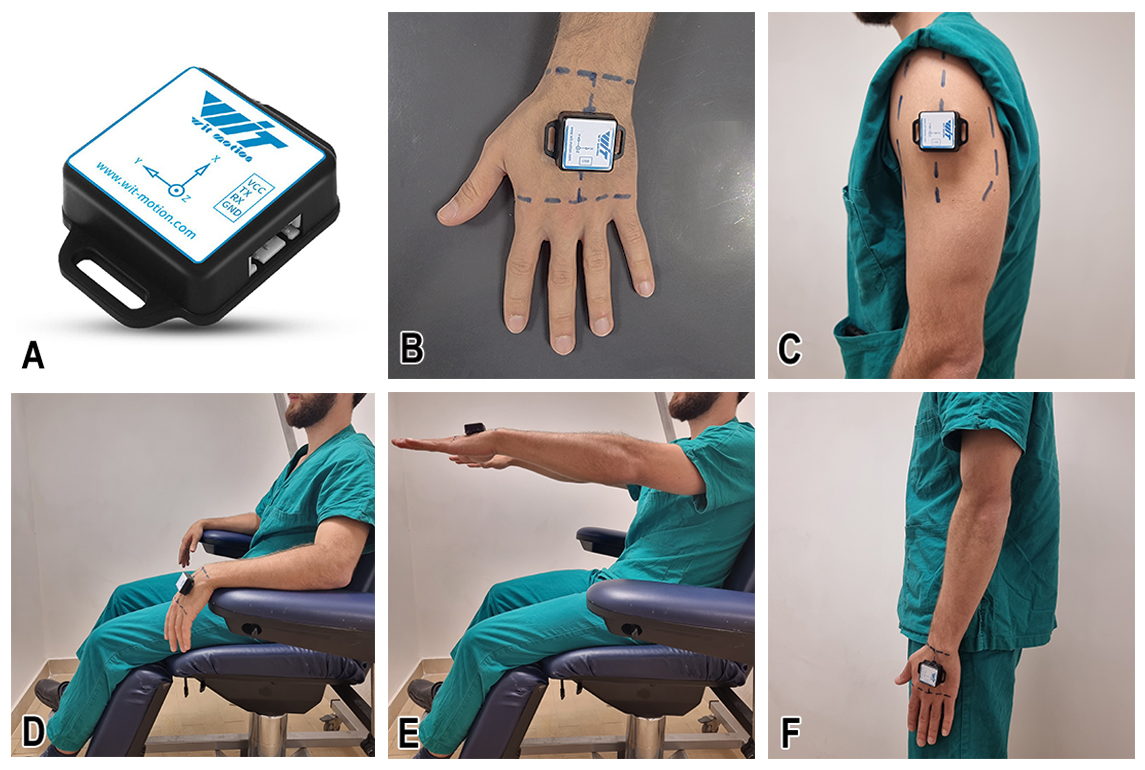

Supplement: Supplementary file 1 — Figure S1. Experimental paradigm. Panel A shows the Inertial Mesurement Unit used in the study (BWT901CL Inertial Measurement Unit by WitMotion Shenzhen Co., Ltd, China). Panel B and C show the anatomical localization of the accelerometer axes for the assessment of tremor on the dorsal side of the hand (B) and on the mid‐deltoid (C). The lower part of the figure illustrates the experimental paradigm, with the conditions used to evaluate rest tremor (D), re‐emergent and postural tremor (E) and tremor during walking (F). [file MDC3-12-226-s005.tiff]

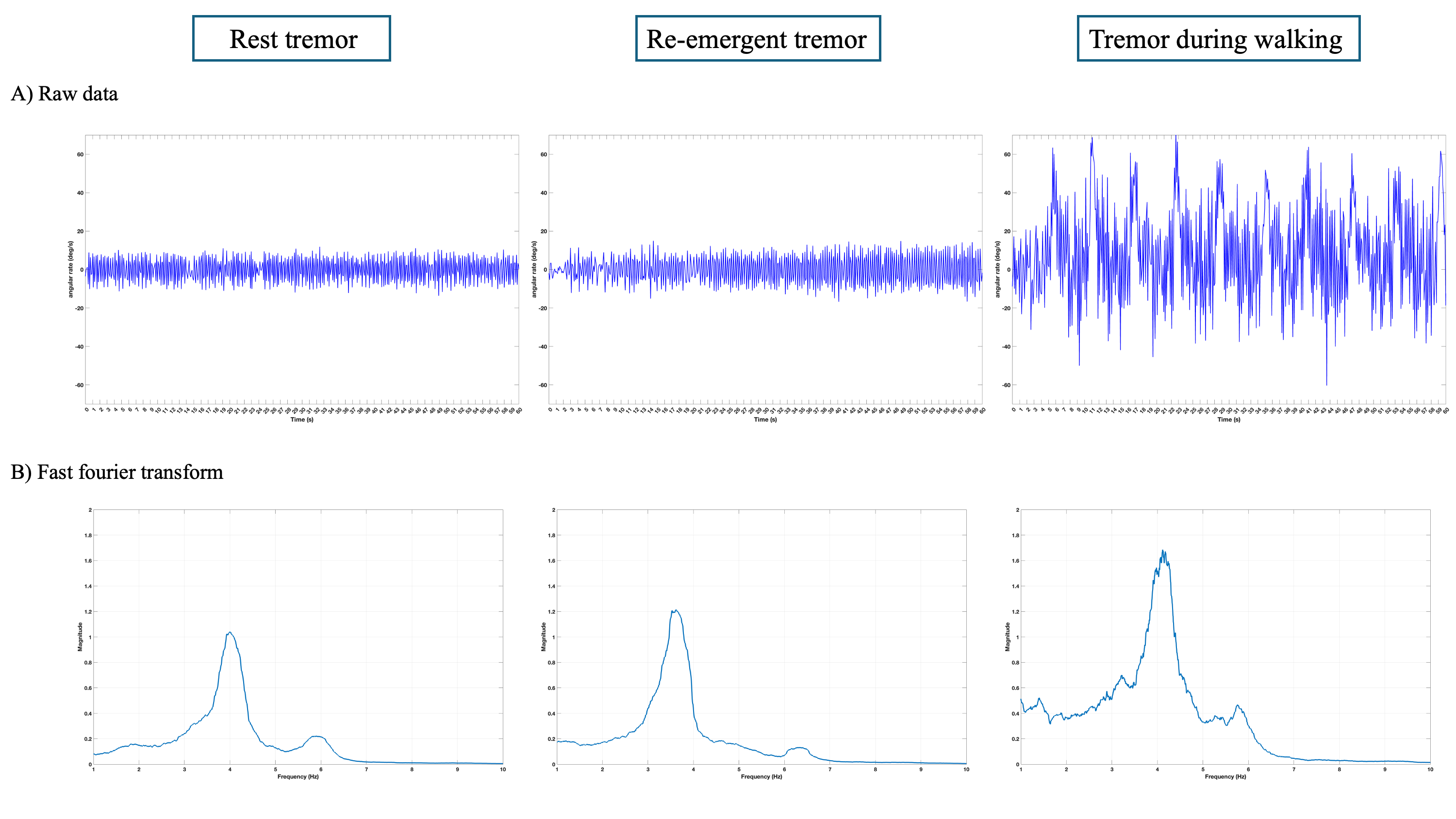

Supplement: Supplementary file 2 — Figure S2. Tremor analysis: raw data and Fast Fourier Transform. Representative data displaying raw measurements (A) and Fast Fourier Transform analysis (B) for a single subject, illustrating conditions of rest tremor, re‐emergent tremor, and tremor during walking. [file MDC3-12-226-s001.tiff]

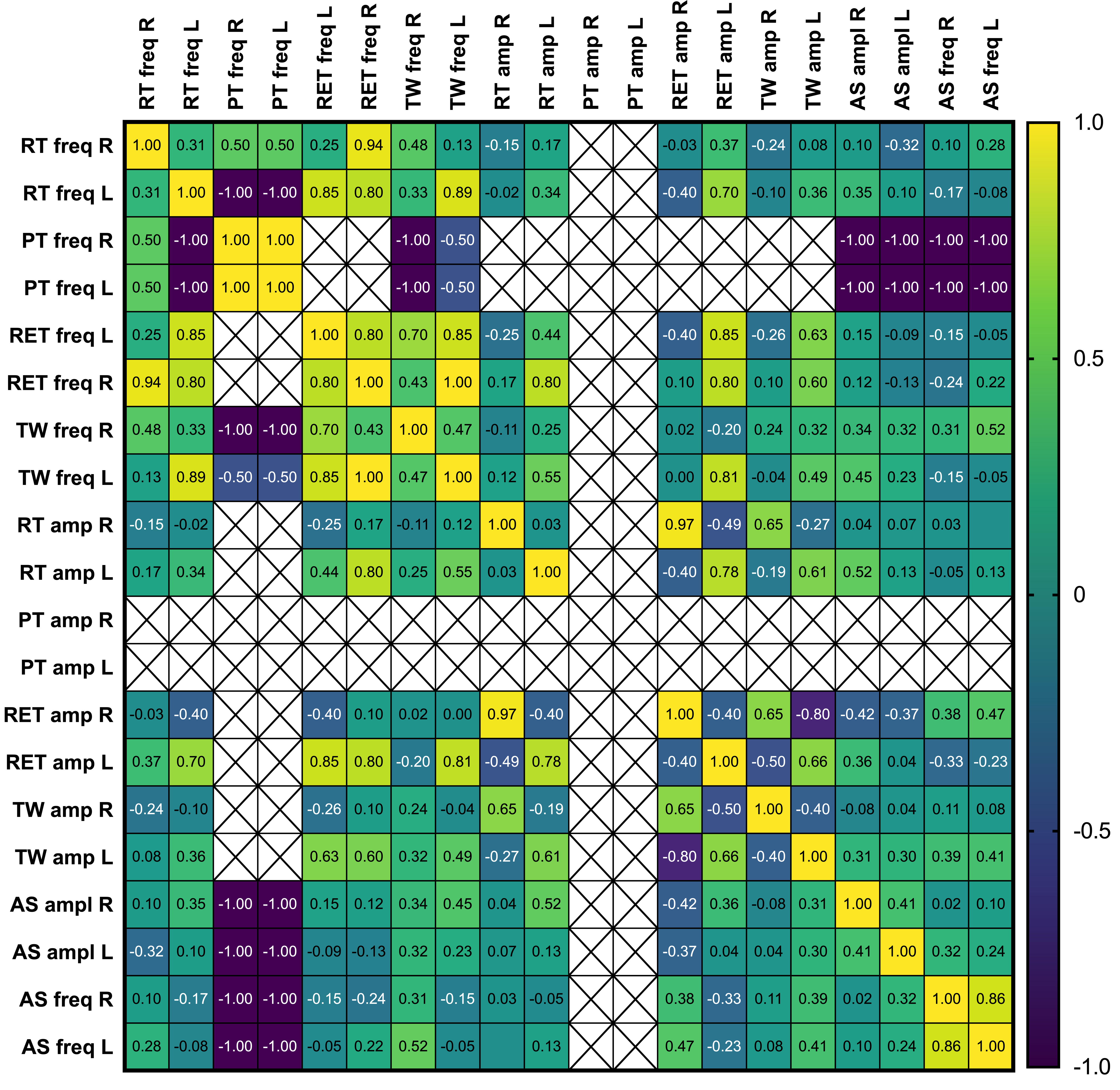

Supplement: Supplementary file 3 — Figure S3. Correlation matrix. The coefficients of correlations between tremor and arm swing parameters (frequency and amplitude) are reported. Abbreviations: RT, resting tremor; PT, postural tremor; RET, re‐emergent tremor; TW, tremor during walking; freq, frequency; amp, amplitude; AS, arm swing. [file MDC3-12-226-s003.tiff]
