# Supplementary material for: Neurophysiological Features of Tremor during Walking in Parkinson's Disease
Source: Mov Disord Clin Pract. 2024 Dec 3;12(2):226–30. doi: 10.1002/mdc3.14293 (PMC11802656; doi:10.1002/mdc3.14293)
Supplement: Supplementary file 4 — Table S1. Neurophysiological characteristics of arm swing movements. [file MDC3-12-226-s002.docx]

**Table S1. Neurophysiological characteristics of arm swing movements**

|  | **Amplitude arm swing right (db/Hz)** | **Amplitude arm swing left (db/Hz)** | **Frequency arm swing (Hz) *** | **Body distribution of TW** |
| --- | --- | --- | --- | --- |
| PD_1 | 1,16 | 12,08 | 1,42 | Left |
| PD_2 | 0,84 | 0,44 | 0,9 | Both |
| PD_3 | 4,79 | 5,45 | 1,75 | Left |
| PD_4 | 7,40 | 1,16 | 1,4 | Both |
| PD_5 | 11,71 | 9,03 | 1,54 | Left |
| PD_6 | 3,83 | 2,41 | 1,51 | Left |
| PD_7 | 16,85 | 15,08 | 1,86 | Both |
| PD_8 | 15,22 | 4,77 | 1,39 | Left |
| PD_9 | 1,95 | 0,43 | 0,8 | Left |
| PD_10 | 6,20 | 13,24 | 2,19 | Left |
| PD_11 | 5,42 | 4,08 | 2,24 | Both |
| PD_12 | 0,74 | 12,44 | 1,85 | Left |
| PD_13 | 24,21 | 9,52 | 1,29 | Right |
| PD_14 | 8,45 | 6,37 | 1,86 | Right |
| PD_15 | 7,54 | 3,30 | 2,22 | Right |
| PD_16 | 17,60 | 16,88 | 1,61 | Both |
| PD_17 | 9,88 | 14,69 | 1,34 | Both |
| PD_18 | 12,88 | 16,80 | 2,34 | Right |
| PD_19 | 10,23 | 8,50 | 1,63 | Right |
| PD_20 | 17,68 | 13,76 | 1,32 | Both |
| PD_21 | 17,80 | 14,75 | 1,73 | Right |
| PD_22 | 9,47 | 3,82 | 1,75 | Right |
| PD_23 | 2,07 | 9,73 | 1,49 | Both |
| PD_24 | 20,49 | 9,89 | 1,81 | Left |
| PD_25 | 21,41 | 3,94 | 1,68 | Left |

*Expressed as the mean frequency of arm swing between the two sides
