# Supplementary material for: Neurophysiological Features of Tremor during Walking in Parkinson's Disease
Source: Mov Disord Clin Pract. 2024 Dec 3;12(2):226–30. doi: 10.1002/mdc3.14293 (PMC11802656; doi:10.1002/mdc3.14293)
Supplement: Supplementary file 5 — Data S1. Additional details on methods, materials, and results are provided. [file MDC3-12-226-s004.docx]

**Supplementary materials**

***Methods and materials***

***Subjects and clinical assessment***

Twenty-five patients diagnosed with idiopathic PD (9F, 16M) were enrolled from the outpatient clinic of Movement Disorders at the Department of Human Neuroscience of Sapienza, University of Rome and IRCCS Neuromed. The diagnosis of PD was made by a movement disorder expert based on the international diagnostic criteria of the Movement Disorders Society (1). The inclusion criteria included clinical evidence of rest tremor and TW, the latter being defined as a tremor that appears immediately or after a time interval in the upper limb when the patient is asked to walk along a straight path with arms relaxed alongside the body (2). The disease stage was assessed using the Hoehn and Yahr scale (H&Y) (3), while the severity of motor symptoms was measured using the Movement Disorder Society-sponsored revision of the Unified Parkinson’s Disease Rating Scale (MDS-UPDRS) part III (4). Finally, the medication regimen was assessed by calculating the Levodopa Equivalent Daily Dose (LEDD) for each patient (5). The protocol was approved by the local ethics committee and performed in accordance with the Declaration of Helsinki.

***Neurophysiological equipment***

A BWT901CL IMU (WitMotion Shenzhen Co.,Ltd, China) was used to evaluate neurophysiological features of tremor and arm swing amplitude. This is a high-precision, wireless sensor device incorporating a 9-axis gyroscope, accelerometer and magnetometer (**Figure S1 A**). For the quantitative analysis of tremor, the device was attached to the dorsum of the participant’s hand. Coordinate axes of the device were required to align with the anatomical reference axes of participant’s hand (**Figure S1 B**). The x-axis was aligned to run from one side of the hand to the other, corresponding to the radial-ulnar direction. The y-axis of the device was aligned to run from the base of the fingers towards the wrist, corresponding to the proximal-distal direction. The z-axis was aligned perpendicular to the surface of the hand. For the quantitative analysis of the amplitude of arm swing during walking, the device was placed on mid-deltoid (**Figure S1 C**). In this case, the x-axis was aligned to run from the posterior side to the anterior side of the muscle, allowing the capture of movement in the forward and backward direction. The y-axis was aligned to run in a cranio-caudal direction, recording upward and downward movements, while the z-axis captured medial-lateral movements. The sampling frequency was set to 200Hz. The IMU data collected were transmitted to a personal computer for storage as .txt files.

***Experimental paradigm***

The experimental session lasts approximately 30 minutes. To standardize data collection and to minimize the influence of dopaminergic therapy on clinical and neurophysiological evaluation, all patients were subjected to a 24 hours withdrawal from dopaminergic treatment and were studied during an OFF-medication period, in the morning, and in the same setting. Initially, demographic data, including age, sex and age at onset as well as LEDD were collected. Clinical features, including disease stage and motor symptoms severity were also collected by using H&Y and MDS UPDRS part III, respectively.

Following clinical evaluation, all patients underwent neurophysiological assessment of tremor and pendular movements amplitude. A wearable wireless gyroscope was placed on the back of the hand (see **Neurophysiological Equipment** and **Figure S1 B**). The hand tremor frequency and amplitude were evaluated under three different conditions, each lasting 1 minute. First, patients were asked to sit on a chair with their forearms resting on armrest (**Figure S1 D**). Rest tremor was assessed by asking the patients to leave their hand hanging from the edge of the armrest and recording only when the tremor had been present for at least 10 seconds (6). Second, patients were instructed to keep their arms in an outstretched position (**Figure S1 E**). Tremor in this position was recorded if it had been present for at least 10 second. Re-emergent tremor was defined as a tremor occurring during the maintenance of the arm outstretched position after any latency period, with a cut-off of 60 seconds, while postural tremor was defined as a tremor occurring without a latency period (7).

Finally, patients were asked to walk for 1 minute along a straight path of 3 meters, making turns as needed (**Figure S1 F**). For each patient, tremor measurements were performed on both hands. The data from both sides were recorded separately. Additionally, to evaluate the amplitude of arm pendular movements, we placed the wearable wireless gyroscope in the proximal portion of each arm (see **Neurophysiological Equipment** and **Figure S1 C**). In the same manner as for the recording of TW, patients were asked to walk for 1 minute along a straight path of 3 meters with turnings. The amplitude of arm pendular movements was measured for both arms.

***Tremor analysis***

The.txt files, which included three-dimensional acceleration and angular velocity raw data were imported into MATLAB (version 9.13.0.2166757 (R2022b), The Mathworks Inc., Natick, MS, USA). The initial preprocessing of the data involved a conversion of time values from a string format (hh:mm:ss.SSS) to seconds. Following this conversion, the raw accelerometer and gyroscope data were plotted against time, to conduct an initial assessment of the data quality and to identify any apparent artefactual change in the signal amplitude patterns over time (**Figure S2 A**). Since all recorded data looked artefact-free from a visual inspection performed by an experienced examiner, all signal was kept for all subjects. We computed the three-dimensional acceleration and angular velocity power spectral density (PSD) between 3 and 12 Hz, on the entire 1-minute long time series, using a Fast Fourier Transform (FFT) (**Figure S2 B**) (8-11). For each gyroscope axis we identified the frequency with the greatest PSD power. The average of the values with the greatest PSD power across the three gyroscope axes was considered as the measure of tremor frequency. Regarding the amplitude of tremor, we calculated the mean of the peak amplitudes across axes (wx, wy, wz), determined at the peak frequencies corresponding to each. Averaging across axes was employed to minimize the impact of potential confounding factors due to hand position / preferential tremor direction. The overall mean peak frequency and mean amplitude of tremors were calculated by averaging the respective measurements taken from both hands for each patient.

***Arm swing amplitude analysis***

The raw data from gyroscope sensors placed on mid-deltoid were processed using MATLAB, to analyse arm swing dynamics during walking. Arm swing, characterized as a rotational movement with a periodicity of around 1-2 Hz (12-13), manifests predominantly in the sagittal plane, with the arm and hand moving in opposite directions (forward and backward) through space. To accurately capture the correlate of arm swing, our analysis focused on rotations about the sagittal axis, that in our experimental setting corresponded to the anatomical X-axis. Movements recorded along this axis reflect indeed forward and backward motions aligned with the body's sagittal plane. This approach was employed to minimize the impact of potential cofounding factors, such as body’s rotational movements that can occur during turns. Frequency components of the arm swing were extracted through the application of the FFT. This analysis facilitated the identification of the predominant frequencies within the arm swing motion, specifically targeting the 0.3-3 Hz range. The magnitudes obtained from the FFT analysis and PSD computation were employed to determine the amplitude of the arm swing.

***Statistical analysis***

We used Prism Graph-Pad version 10.1.1 (Boston, USA) for the statistical analysis. Continuous variables are reported as means ± standard deviations. The Shapiro–Wilk test was used to determine whether all variables fitted a normal distribution. Depending on the data distribution, parametric and non-parametric tests were used to evaluate potential differences in terms of frequency and amplitude between tremors, as appropriate. Spearman rank correlation coefficient was calculated to test the association of several characteristics of various tremors. Specifically, we analyzed potential correlations between the mean peak frequencies, the mean amplitude both within each side and between the two sides of different tremors (rest tremor, TW and re-emergent). We also assessed the correlations between the mean amplitudes or mean peak frequencies of the tremors and the magnitude of arm swing. P values < 0.05 were considered as significant. Tukey correction was used to correct for multiple comparisons.

**Results**

***Demographic and clinical characteristics of PD patient***

Twenty-five PD patients participated at the study. The mean age was 67.4 ± 12 years, while the mean age at onset was 61.5 ± 12 years. The mean disease duration was 5.8 ± 3.5 years. The mean H&Y stage was 2.32 ± 0.69. The mean MDS-UPDRS Scale part III total score was 22.5 ± 9.4, while the mean LEDD was 370.4 ± 215.7.

***Body distribution of various types of tremors***

Rest tremor appeared bilaterally in 10 out of 25 patients, and unilateral in 15 out of 25 patients (right side 7 patients, left side 8 patients). Re-emergent tremor was present in 18 out of 25 patients, of which 15 patients had unilateral re-emergent tremor (right side 5 patients, left side 10 patients). TW appeared bilaterally in 8 out of 25 patients, and unilaterally in 17 of 25 patients (right side 7 patients, left side 10 patients). Postural tremor was present in only 3 patients, always bilaterally. The body distribution of TW matched that of rest tremor in 23 out of 25 patients. Specifically, all 15 patients with unilateral rest tremor had a unilateral TW on the same side. Of the 10 patients with bilateral rest tremor, 8 had a bilateral TW, and the remaining 2 patients had bilateral rest tremor with TW only in the left upper limb. The body distribution of TW also matched that of re-emergent tremor in 14 out of 18 patients. In particular, 2 out of 3 patients with bilateral re-emergent tremor had a bilateral TW, while 12 out of 15 patients with unilateral re-emergent tremor had an unilateral TW on the same side. Of the remaining patients, one subject with bilateral re-emergent tremor had TW only on the left side, while the other 3 patients with unilateral re-emergent tremor had bilateral TW.

***Tremor frequency analysis***

The overall mean peak frequency, calculated by averaging the mean peak frequency from both sides, for resting tremor was 4.1 ± 0.6 Hz, for re-emergent tremor it was 4.3 ± 0.6 Hz, for TW it was 4.3 ± 0.6 Hz, and for postural tremor it was 4.9 ± 0.6 Hz (see also **Table 1**). A two-way repeated measure ANOVA was conducted to analyse the differences in frequency between different types of tremors (rest, re-emergent, postural and TW) and across different body sides (right, left). ANOVA analysis showed that while there was a significant main effect of TREMOR TYPE (F = 5.651, DFn = 3, DFd = 44; p = 0.0023), no significant effect were found for SIDE (F = 0.2840, DFn = 1, DFd = 44; p = 0.5968) or the interaction between SIDE and TREMOR TYPE (F = 1.422, DFn = 3 , DFd = 44; p = 0.2490). Despite the significant main effect of TREMOR TYPE, subsequent post-hoc analysis did not reveal any significant differences (**Figure 1A**). Spearman’s correlation coefficient showed a positive correlation between the mean peak frequencies of rest tremor and those of TW (r = 0.512; p =0.04) and re-emergent tremor (r = 0.819; p = 0.007), all on the right side. Similarly, on the left side, a positive correlation was also found between the mean peak frequencies of rest tremor and those of TW (r = 0.860; p = 0.000005) and re-emergent tremor (r = 0.811; p = 0.000766). Additionally, the mean peak frequencies of TW were positively correlated with those of re-emergent tremor (r = 0.743; p = 0.001).

***Amplitude of different types of tremors***

The global mean amplitude, calculated by averaging the mean amplitude from both sides, for resting tremor was 18.3 ± 11.6 dB/Hz, for re-emergent tremor it was 16.6 ± 13.2 dB/Hz, for TW it was 27.3 ± 8.5 dB/Hz, and for postural tremor it was 18.5 ± 16.3 dB/Hz (see also **Table 1**). A two-way repeated measure ANOVA, conducted to analyse the potential differences in amplitude across different types of tremors (resting, re-emergent, postural and TW) and between body sides (right, left), revealed that there was a significant main effect of TREMOR TYPE (F = 21.61, DFn = 3, DFd = 50; p < 0.0001). Conversely, no significant effect was found for SIDE (F = 0.003543, DFn = 1, DFd = 32; p = 0.9529) or the interaction between SIDE and TREMOR TYPE (F = 0.8444, DFn = 3, DFd = 50; p = 0.4761). The post-hoc analysis showed significant amplitude differences between rest and TW on both sides, with rest tremor showing lower amplitudes on the right side (mean difference = -9.918; CI = -15.53 to -4.307; adjusted p value = 0.0008) and on the left side (mean difference = -8,203; CI = -14.21 to -2.195; adjusted p value = 0.0065). Also, re-emergent tremor amplitude was lower than TW amplitude on the left side (mean difference = -12.4; CI = -19.49 to -5.225; adjusted p value = 0,0014) (**Figure 1B**). Finally, Spearman’s correlation coefficient showed a positive correlation between the mean amplitude of rest tremor and that of TW (r = 0.725; p = 0.002212) and re-emergent tremor (r = 0.963; p = 0.000032), as well as between the mean amplitude of TW and that of the re-emergent tremor (r = 0.733; p = 0.02) all on the right side. Similarly, on the left side, a positive correlation was also found between the mean amplitude of resting tremor and those of TW (r = 0.739; p = 0.001) and re-emergent tremor (r = 0.921; p = 0.00002). Also, the mean amplitude of TW was positively correlated with that of the re-emergent tremor (r = 0.723; p = 0.008). No significant correlation was found between the amplitudes of different types of tremors and the most affected side in terms of bradykinesia

**References**

1. Postuma RB, Berg D, Stern M, et al. MDS clinical diagnostic criteria for Parkinson’s disease: MDS-PD Clinical Diagnostic Criteria. Mov Disord 2015 Oct;30(12):1591–601.
2. Costanzo M, Cutrona C, Leodori G, et al. Distal Upper Limb Tremor during Walking in Parkinson’s Disease. Mov Disord Clin Pract 2023 Aug;10(8):1198–202.
3. Hoehn MM, Yahr MD. Parkinsonism: onset, progression and mortality. Neurology. 1967 May;17(5):427–42.
4. Antonini A, Abbruzzese G, Ferini-Strambi L, et al. Validation of the Italian version of the Movement Disorder Society--Unified Parkinson’s Disease Rating Scale. Neurol Sci 2013 May;34(5):683–7.
5. Jost ST, Kaldenbach M, Antonini A, et al. Levodopa Dose Equivalency in Parkinson’s Disease: Updated Systematic Review and Proposals. Mov Disord 2023 Jul;38(7):1236–52.
6. Leodori G, Belvisi D, De Bartolo MI, et al. Re‐emergent Tremor in Parkinson’s Disease: The Role of the Motor Cortex. Mov Disord 2020 Jun;35(6):1002–11.
7. Belvisi D, Conte A, Bologna M, et al. Re-emergent tremor in Parkinson’s disease. Parkinsonism & Related Disorders 2017 Mar;36:41–6.
8. Bartolić A, Šantić M, Ribarič S. Automated tremor amplitude and frequency determination from power spectra. Computer Methods and Programs in Biomedicine 2009 Apr;94(1):77–87.
9. Martinez Manzanera O, Elting JW, Van Der Hoeven JH, Maurits NM. Tremor Detection Using Parametric and Non-Parametric Spectral Estimation Methods: A Comparison with Clinical Assessment. PLoS ONE 2016 Jun 3;11(6):e0156822.
10. Timmer J, Lauk M, Deuschl G. Quantitative analysis of tremor time series. Electroencephalography and Clinical Neurophysiology 1996 Oct;101(5):461–8.
11. Vial F, Kassavetis P, Merchant S, Haubenberger D, Hallett M. How to do an electrophysiological study of tremor. Clinical Neurophysiology Practice 2019;4:134–42.
12. Warmerdam E, Romijnders R, Welzel J, Hansen C, Schmidt G, Maetzler W. Quantification of Arm Swing during Walking in Healthy Adults and Parkinson’s Disease Patients: Wearable Sensor-Based Algorithm Development and Validation. Sensors 2020 Oct 21;20(20):5963.
13. Warmerdam E, Romijnders R, Hansen C, Elshehabi M, Zimmermann M, Metzger FG, et al. Arm swing responsiveness to dopaminergic medication in Parkinson’s disease depends on task complexity. NPJ Parkinsons Dis 2021 Oct 5;7(1):89.
